# Integrating Oral Health Into Occupational Therapy: Knowledge, Practices, Barriers and Facilitators—A Systematic Review

**DOI:** 10.1155/ijod/2007891

**Published:** 2026-10-12

**Authors:** Ming Nam Tse, Kristy Coxon, Navira Chandio, Kh Shafiur Rahaman, Shruti Nair, Ajesh George, Kanchana Ekanayake, Rosalind Bye, Grace Wong, Carol Tran, Maria O’Reilly, Amit Arora

**Affiliations:** ^1^ School of Health Sciences Western Sydney University Penrith New South Wales 2751 Australia westernsydney.edu.au; ^2^ Health Equity Across the Lifecourse Research Lab Campbelltown New South Wales 2560 Australia; ^3^ Translational Health Research Institute Western Sydney University Campbelltown New South Wales 2560 Australia westernsydney.edu.au; ^4^ School of Medicine Faculty of Health Western Sydney University Campbelltown Campus, Locked Bag Penrith New South Wales Australia westernsydney.edu.au; ^5^ Bangladesh Academy of Dietetics and Nutrition (BADN) Dhaka Bangladesh; ^6^ Oral Health Services Western Sydney Local Health District NSW Health Westmead New South Wales 2145 Australia nsw.gov.au; ^7^ Australian Centre for Integration of Oral Health (ACIOH) School of Nursing and Midwifery Western Sydney University Liverpool New South Wales 1871 Australia westernsydney.edu.au; ^8^ Sydney Dental School Faculty of Medicine and Health The University of Sydney Westmead New South Wales 2145 Australia sydney.edu.au; ^9^ School of Nursing University of Wollongong Wollongong New South Wales 2522 Australia uow.edu.au; ^10^ Ingham Institute for Applied Medical Research Liverpool New South Wales 1871 Australia inghaminstitute.org.au; ^11^ University of Sydney Library The University of Sydney Camperdown New South Wales 2006 Australia sydney.edu.au; ^12^ Oral Health Services Primary and Community Health Northern Sydney Local Health District NSW Health North Ryde New South Wales 2113 Australia; ^13^ School of Nursing and Midwifery Western Sydney University Penrith New South Wales 2751 Australia westernsydney.edu.au; ^14^ Private Practice Brisbane Queensland 4000 Australia; ^15^ School of Health, Medical and Applied Sciences CQUniversity Bundaberg Queensland Australia cqu.edu.au; ^16^ Discipline of Child and Adolescent Health Faculty of Medicine and Health The Children’s Hospital at Westmead Clinical School The University of Sydney Westmead New South Wales 2145 Australia sydney.edu.au; ^17^ Oral Health Services Sydney Local Health District and Sydney Dental Hospital NSW Health Surry Hills New South Wales 2010 Australia; ^18^ Aureolin Research, Consultancy and Expertise Development Foundation 7 Avenue 5 Dhaka 1216 Bangladesh; ^19^ Young and Resilient Research Centre Western Sydney University Locked Bag 1797 Penrith New South Wales 2751 Australia westernsydney.edu.au

**Keywords:** barriers, facilitators, integration, knowledge, occupational therapists, oral health, practices, systematic review

## Abstract

**Background:**

Oral health is essential for overall wellbeing and daily functioning, yet its integration into occupational therapy remains limited. Despite the profession’s potential to promote oral health through person‐centred, occupation‐based approaches, evidence of this integration is scarce. This systematic review explores occupational therapists’ (OTs) knowledge and practices in oral health care and examines the barriers and facilitators to incorporating oral health care into their clinical practice.

**Methods:**

A comprehensive search was conducted across four databases: MEDLINE (Ovid), CINAHL (EBSCO), SCOPUS and EMBASE (Ovid). Studies were deemed eligible if they reported on OTs’ knowledge and practices, as well as the barriers and facilitators to incorporating oral health into clinical practice. Two reviewers independently screened studies, extracted data and resolved discrepancies through team consultation. We assessed methodological quality using the Joanna Briggs Institute Critical Appraisal Checklist for Qualitative Research. We performed thematic analysis and reported the results in accordance with the Preferred Reporting Items for Systematic Reviews and Meta‐Analyses guidelines.

**Results:**

Of the 14,138 records screened, three qualitative studies met the inclusion criteria. While these studies highlighted OTs’ recognition of oral health’s impact on daily living and their awareness of person‐related challenges to oral hygiene, the relatively small number of included studies limits the generalisability of these findings. Reported practices included assessment, education, environmental modifications, and sensory strategies. Facilitators included prior therapist training, family‐centred care and access to resources for oral health intervention. Barriers included limited oral health knowledge, insufficient training, systemic service gaps, lack of oral healthcare supplies and other resource constraints.

**Conclusion:**

This review suggests preliminary insights that OTs could potentially play a meaningful role in promoting oral health but are constrained by barriers at individual, organisational and systemic levels. Strengthening workforce training, interprofessional collaboration and policy support may help address oral health needs within occupational therapy practice.

## 1. Introduction

Oral health is a multidimensional concept encompassing not just the absence of oral disease but also functional dentition, optimal oral functions and overall quality of life [1]. Dental caries (tooth decay) and periodontal disease (gum disease) are among the most prevalent chronic conditions globally, posing substantial public health challenges due to their high prevalence, long‐term consequences and costly treatment demands [2]. According to the Global Oral Health Status Report by the World Health Organisation (WHO), oral diseases affect over 3.5 billion people, with the burden disproportionately concentrated in low‐ and middle‐income countries and among socially disadvantaged populations in high‐income countries [3, 4].

In 2019, worldwide spending on oral diseases totalled $387 billion across 194 countries, averaging $50 per person and accounting for 4.8% of total health care expenditures. Productivity losses added an estimated $42 per capita, totalling approximately $323 billion globally [5]. In Australia alone, poor oral health accounted for 4.5% of all non‐fatal disease burden in 2022 [6], and the dental expenditure reached AU$11.1 billion in 2020–2021, comprising 7.4% of total disease‐related health care spending [6].

Globally, there are significant disparities in the dental care expenditure. In low‐income countries, the average annual spending on dental care per capita is US$0.52, while in high‐income countries, the average cost is US$260, a 500‐fold difference [5]. This highlights the substantial economic burden oral conditions impose on individuals and society, disproportionately impacting socioeconomically disadvantaged groups [7] as well as people with disabilities who tend to have poorer oral health outcomes compared to healthy individuals [8, 9]. Limited oral health literacy, cost, and inequitable access to dental care further exacerbate these disparities [10–12]. Poor oral health is closely linked to compromised nutrition, chronic conditions, including diabetes and cardiovascular disease, and reduced quality of life [13]. By 2050, severe periodontitis is expected to rank among the leading causes of disability, as measured by age‐standardised years lived with disability (YLD) rates [14].

To address these issues, international and national policy initiatives recommend preventive, interdisciplinary and equitable approaches to oral health. The Bangkok Declaration on Oral Health in 2024 emphasises the importance of incorporating oral health promotion into broader health systems, particularly through non‐dental health professionals [15] as health professionals in primary care and allied health are increasingly recognised as key partners in delivering oral health promotion and preventive care [16, 17].

Occupational therapists (OTs) are well‐positioned to contribute to this evolving landscape due to their comprehensive, person‐centred and function‐oriented approach to wellbeing. OTs support individuals in managing daily routines and self‐care tasks, including oral hygiene, by addressing physical, sensory, cognitive and environmental barriers [18]. Research has demonstrated the effectiveness of OTs in delivering oral hygiene interventions in diverse settings, such as aged care facilities, post‐stroke rehabilitation, and paediatric care for children with sensory needs [19, 20]. With their expertise in adapting tasks and environments, OTs are uniquely suited to improve oral health outcomes through sustainable, person‐specific strategies. Despite this potential, oral health remains an under‐represented domain within the occupational therapy literature, education, and practice. Current research on the knowledge and practices of OTs related to oral health is limited, and even less attention has been paid to the barriers and facilitators that shape their engagement in this domain [21]. This is concerning given the clear relationship between oral health and systemic health outcomes, especially among vulnerable populations [13].

To date, the available evidence on oral health care in occupational therapy practice has not been synthesised. Understanding the knowledge base and clinical approaches of OTs, as well as contextual barriers, is critical for developing effective training programmes, informing health policy, and guiding future practice and research. This review seeks to address these gaps by consolidating the evidence on OT involvement in oral health care. Specifically, it aims to provide an in‐depth understanding of knowledge and practices and to identify key facilitators and barriers influencing the integration of oral health into the occupational therapy practice.

## 2. Materials and Methods

This systematic review was conducted in accordance with the Joanna Briggs Institute (JBI) methodology for evidence synthesis [22] and reported in line with the Preferred Reporting Items for Systematic Reviews and Meta‐Analyses (PRISMA 2020) guidelines (Table S1) [23] and the Synthesis Without Meta‐analysis (SWiM) reporting framework [24]. The review protocol was registered with the International Prospective Register of Systematic Reviews (PROSPERO; CRD42024522136) [25], and the protocol of this review has been published previously [26].

### 2.1. Eligibility Criteria

The inclusion and exclusion criteria were formulated according to the population, concept and context (PCC) framework [27].

#### 2.1.1. Population (P)

OTs work in any context, including but not limited to community health centres, hospitals, disability services, aged care services, schools and private practices.

#### 2.1.2. Concept (C)

This systematic review explored four phenomena of interest. First, we investigated oral health knowledge and oral health practices—exploring the level of knowledge about oral health and how this knowledge translates into OTs’ daily professional practices. We then identify barriers to integrating oral health into occupational therapy practices and finally focus on facilitators—elements that promote or ease its integration.

#### 2.1.3. Context (C)

The systematic review focused on oral health, covering a wide range of related topics. This includes, but is not limited to, dental health, oral hygiene practices, dental care services, prevention and management of dental caries, gum disease, oral diseases and factors influencing dental visits.

### 2.2. Information Sources and Search Strategy

The electronic databases searched included Medline (Ovid), CINAHL (EBSCO), SCOPUS and EMBASE (Ovid). We searched all databases from inception to 21st January 2025, with a top‐up search on 5th September 2026. Studies using quantitative, qualitative and mixed‐methods research designs, published in English in peer‐reviewed journals, were included in this review with no restrictions on the publication date or region.

The initial search terms were generated using the PCC framework and presented in a logic grid. To increase sensitivity and ensure satisfactory search retrieval, a combination of Medical Subject Headings (MeSH) terms and keywords was employed, along with Boolean operators, phrase searching, spelling variations, and truncation. Two reviewers (Ming Nam Tse and Navira Chandio) pre‐tested the search strategy in Medline (Ovid), with guidance from a Health Sciences Librarian. Once the Medline search was completed, the strategy was adapted to the syntax and subject headings of the other databases. A detailed search strategy is available in Table S2.

### 2.3. Study Selection

All studies identified through database and manual searches were first imported into EndNote X9 reference management software to remove duplicates [28]. The deduplicated records were then imported into Covidence systematic review software for screening [29]. Two reviewers (Ming Nam Tse and Navira Chandio) independently screened the titles and abstracts against the eligibility criteria, retrieving the full texts for clarification when necessary. Studies that met the inclusion criteria or had unclear eligibility underwent a full‐text review. The full texts of all eligible studies were assessed independently by two reviewers to determine final inclusion. Study authors were contacted if further information was required. Any disagreements regarding study inclusion or exclusion were resolved through consultation with a third reviewer (Amit Arora). Reasons for excluding studies at the full‐text review stage were documented (Table S3), and the selection process is presented in a PRISMA flow diagram (Figure 1).

**Figure 1:**
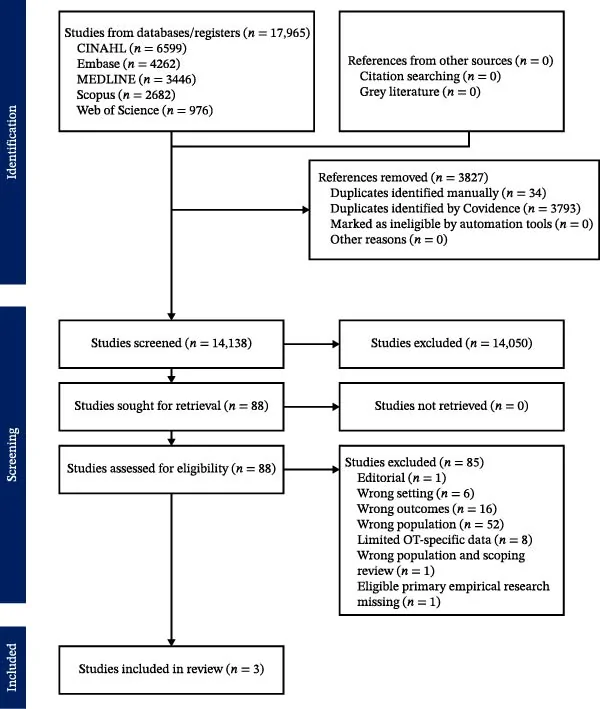
PRISMA flow diagram showing the selection of studies for the systematic review.

### 2.4. Data Extraction

A standardised data extraction form was used to capture relevant information from each included study systematically. Before commencing full extraction, a pilot test was conducted on three studies to ensure the tool’s consistency and reliability. Two reviewers (Ming Nam Tse and Amit Arora) independently extracted the data, and any discrepancies were resolved through team discussion.

The data were extracted based on the following characteristics:

Study reference—citation details including authors, title and publication year.

Study aim—the primary objective or research question of the study.

Setting and design, comprising:

Study design—type of study (qualitative, quantitative or mixed‐methods).
Location—country and city/region where the study was conducted.
Methodology—approach used for data collection and analysis.
Sampling method—recruitment strategies and sampling techniques employed.

Participants—OTs working in clinical settings such as hospitals, community health services, long‐term care facilities or private practices.

Outcomes measured—knowledge and practices related to oral health, along with enablers and barriers to integration within the occupational therapy practice.

Study limitations—any methodological limitations or potential biases as reported by the study authors.

### 2.5. Data Synthesis

A narrative synthesis approach was used to analyse key findings. The qualitative studies were independently analysed by three reviewers (Ming Nam Tse, Amit Arora and Kristy Coxon) using Quirkos software [30]. In the first step, themes and sub‐themes were developed from the extracted data. The coded data within these sub‐themes were then placed into one of four categories: knowledge, practice, facilitator or barrier. A fourth reviewer (Navira Chandio) independently reviewed and validated the emerging thematic framework to ensure consistency and credibility of the interpretation. This was followed by iterative discussions among the research team to refine and reach consensus on the final analytical themes, yielding deeper insights into the integration of oral health into the occupational therapy practice.

### 2.6. Quality Assessment

The methodological quality of the included studies was evaluated using the JBI Critical Appraisal Checklist for Qualitative Research, which comprises 10 items rated as ‘yes’, ‘no’ or ‘unclear’ [31]. Each study received an overall rating of ‘include’, ‘exclude’ or ‘seek further information’ [31]. One reviewer (Ming Nam Tse) independently appraised all three studies; each study was subsequently cross‐checked by a second reviewer (Amit Arora, Navira Chandio or Kristy Coxon). Any disagreements were resolved through discussion among the review team (Ming Nam Tse, Amit Arora, Navira Chandio and Kristy Coxon). Authors were contacted for clarification on the missing or unclear information. The risk of bias for each study is summarised in the tables (Table S4) and described narratively.

### 2.7. Assessment of Confidence in the Review Findings

The Grading of Recommendations Assessment, Development and Evaluation Confidence in the Evidence from Reviews of Qualitative Research (GRADE‐CERQual) approach was used to assess confidence in each of the review findings generated through the thematic synthesis [32]. The GRADE‐CERQual assesses the extent to which each synthesised review finding is a reasonable representation of the phenomenon of interest based on four components: (i) methodological limitations, (ii) coherence, (iii) adequacy of data and (iv) relevance.

Each of the four components was assessed independently for every review finding and judged as having no or minor, moderate or serious/very serious concerns. These component‐level judgements were then considered together to reach an overall assessment of confidence in each finding, categorised as high, moderate, low or very low, following the approach outlined by Lewin et al. [32]. Assessments were conducted independently by Amit Arora and Navira Chandio, with disagreements resolved through discussion. The GRADE‐CERQual assessment is presented in Table S5, and a narrative summary is provided in Section 3.5.

## 3. Results

### 3.1. Study Selection

A total of 17,965 records were identified through comprehensive searches of the four electronic databases. After removing duplicates, 14,138 unique records remained for title and abstract screening. Of these, 14,050 were excluded based on the eligibility criteria, and 88 full‐text articles were retrieved for further assessment. After a full‐text review, 85 studies were excluded for not meeting the inclusion criteria. Although the search strategy was designed to include qualitative, quantitative and mixed‐methods studies, only three qualitative studies met the review eligibility criteria and were ultimately included in the final synthesis. Details of the excluded studies and reasons for exclusion are provided in Table S3. The PRISMA flow diagram (Figure 1) illustrates the full selection process.

### 3.2. Study Characteristics

All included studies employed qualitative research methods, primarily using focus groups, with one study also conducting semi‐structured interviews. The studies were published in English between 2017 and 2023 and conducted in high‐income countries—Sweden, Ireland and Singapore. Two studies reported using structured interview guides, while one adopted an open discussion format. Participants were OTs from various settings, such as stroke units, geriatric wards and community health care, with extensive professional experience and multidisciplinary backgrounds (Table 1).

**Table 1:** Characteristics of the included studies.

Author, year	Country	Study aim	Study setting and methods	Participants	Outcomes			
					Knowledge	Practices	Facilitators	Barriers
Curtin et al. 2023 [19]	Ireland	To explore the attitudes, facilitators and barriers as perceived by health care professionals in providing oral care for inpatients with dysphagia post‐stroke.	Stroke Unit, Cork University Hospital. Qualitative: three focus groups were conducted.	17 healthcare professionals included: dietician (1), OT (1), OT assistant (1) health care assistants (2), nurses (4), physiotherapists (3), nursing students (2), speech language therapists (2), doctor (1).	Limited knowledge: Lack of training and confidence in oral health care delivery creates uncertainty.	—	Sufficient resources: Accessible tools and supplies, such as assessment instruments and toothbrushes, support oral health care delivery.	Limited oral health literacy: Oral health care is undervalued in rehabilitation and not prioritised in daily routines. Person‐related challenges: Resistance, pain and dependence on assistance hinder effective oral health care. Limited knowledge: Lack of training and confidence in oral health care delivery creates uncertainty. Systemic barriers: Absence of clear guidelines and competing priorities impede care. Resource limitations: Inadequate access to tools and dental services disrupts effective intervention.
Grönbeck Linden et al. 2017 [33]	Sweden	To identify factors that may affect an older adult’s ability to perform oral hygiene self‐care.	Gothenburg, Sweden, and its vicinity. Qualitative: four focus groups were conducted, and content analysis was performed to analyse the study data.	23 participants: dental hygienists (5), assistant nurses (6), OTs (5), and elderly individuals (7).	Knowledge of oral health care conditions: The therapists recognised toothbrushing as a complex, multifaceted task involving motor, sensory, and cognitive processes. This recognition underscored the need for adaptations and targeted support for individuals.	Environmental modifications: Adapting bathrooms to enable older adults to sit comfortably during oral hygiene tasks. Ensuring access to mirrors to support independence and accuracy in oral health care routines.	Understanding of personal factors: Knowledge and experience were identified as motivational factors essential for maintaining oral hygiene. Emphasis was placed on establishing oral hygiene habits early in life to ensure consistency.	Person‐related challenges: Emotional distress, sensory‐motor limitations and cognitive deficits hinder oral hygiene. Additionally, people often lack initiative, even when they have the physical capability and knowledge.
Junnarkar et al. 2022 [20]	Singapore	To determine perceived issues, barriers, and potential solutions to dental care from the perspective of OTs and speech therapists for children with autism spectrum disorder (ASD) in Singapore.	Dental hospital, community clinic, and Early Intervention Programme for Infants and Children (EIPIC) centre, Singapore.	12 participants: OTs (7) and speech therapists (5).	Understanding of oral health conditions and habits: OTs and speech therapists identified toothbrushing issues, oral pathology and inadequate toothpaste use. Identifying reasons for difficulties in home oral health care: OTs recognised issues affecting oral hygiene maintenance in children with autism—for example, sensory sensitivities and functional difficulties like spitting, etc.	Assessment: Conducted formal assessments to identify sensory issues such as taste sensitivity, toothbrush intolerance and oral habits like mouthing or biting. Recognised broader sensory challenges, including aversion to face‐washing and picky eating. Environmental modification: Recommended alternative toothbrushing locations and adjusted environments, such as sinks and mirrors at suitable heights. Advocated for sensory‐friendly dental waiting areas with calming features such as specialised lighting and noise cancellation. Education: Used modelling, visual schedules, pretend play and desensitisation techniques to teach oral health care routines. Prepared children for dental visits with social stories, videos and toy dental tools. Sensory processing strategies: Suggested alternative aids (e.g., finger gloves, electric toothbrushes) for sensory‐sensitive children. Employed sensory interventions such as oral massage, deep pressure techniques and weighted vests. Utilised sensory tools like swings and beanbags to help children adjust to dental chairs.	Understanding of personal factors: Acknowledged the stress associated with dental visits for parents of children with autism spectrum disorder and emphasised the need for individualised planning. Adequate training: Basic sensory integration therapy is part of the occupational therapy curricula, with further certifications for those specialising in paediatrics. Family‐centred practice: Emphasised the involvement of caregivers in consultations and treatment planning to ensure consistent and effective implementation of oral health strategies. Multidisciplinary team involvement: Advocated for interdisciplinary collaboration to develop individualised plans for children with autism, ensuring successful dental visits that address specific needs. Sufficient tools and resources: Encouraged exploration of tools and techniques to address sensory processing and oral‐motor challenges, including teaching children to spit and facilitating dental treatment.	Limited oral literacy: Toothbrushing was often not prioritised by parents or was missed due to caregiver absence. Parental confusion regarding fluoridated toothpaste and its role in caries prevention. Person‐related challenges: Difficulties with toothpaste texture, spitting and gargling are often linked to sensory and motor issues in children with autism. Parental avoidance of fluoride toothpaste due to concerns about ingestion. Insufficient knowledge among therapists: Therapists were unsure of toothpaste recommendations for sensory issues and optimal fluoride levels. Lack of clarity when guiding parents about fluoride use. Systemic barriers: Absence of clear referral pathways to dental care, leaving parents to find dentists independently. Resource limitations: Limited engagement in pre‐dental visit preparation due to resource constraints.

### 3.3. Study Findings

The findings are organised across four thematic domains: (1) knowledge of OT’s on oral health care, (2) current practices of OT’s in providing oral health care, (3) perceived enablers for OT’s in providing oral health care and (4) Perceived Barriers for OTs in providing oral health care delivery.

#### 3.3.1. Knowledge of OT’s on Oral Health Care

##### 3.3.1.1. Understanding of Oral Health Conditions and Habits

Three studies explored OT knowledge of oral health conditions and related habits within their respective populations [20, 33]. Curtin et al. [19] reported that OTs had limited knowledge, while Junnarkar et al. [20] reported that OTs identified issues such as gross oral pathology, inappropriate or inconsistent use of fluoridated toothpaste, and behaviours associated with sensory sensitivities. These included hypersensitivity to toothbrush bristles, dislike of specific toothpaste flavours, swallowing toothpaste, and biting the toothbrush. Such behaviours were commonly reported in children with autism spectrum disorder (ASD) and in individuals with oral sensory processing challenges [20]:

Difficulty in accepting the texture or taste of toothpaste (e.g., mint) was another reason for the avoidance of toothpaste or the use of milder‐tasting, non‐optimally fluoridated toothpaste. [20]

##### 3.3.1.2. Recognition of Person‐Related Factors

OTs demonstrated detailed knowledge of personal factors influencing oral hygiene [20, 33]. Grönbeck Linden et al. [33] identified physical limitations such as poor upper body control and balance, which hindered actions like leaning forward to spit, and hearing impairments, which reduced the ability to follow verbal instructions.

The person may not have a good enough sense of balance and cannot control the upper body, cannot lift the arm because then they fall over. Leaning forward to spit may also be difficult. OT [33]

##### 3.3.1.3. Understanding the Complexity of Oral Health Care Activities

All studies acknowledged that oral hygiene tasks require fine coordination between motor, sensory and cognitive systems [20, 33]. This complexity was particularly apparent in populations with neurological or developmental conditions, where challenges could arise at any stage of the task.

The participants pointed out different factors of significance for performing oral hygiene that showed that toothbrushing is a complex activity. This complexity was especially underlined by the occupational therapists. [33]

#### 3.3.2. Current Practices of OT’s in Providing Oral Health Care

Two studies examined how OTs promoted independence in oral hygiene [20, 33]. Reported practices were categorised into four domains: assessment, environmental modifications, educational strategies and sensory‐based approaches.

##### 3.3.2.1. Assessment

OTs incorporated formal and informal assessments to identify oral health care challenges [20]. These included structured caregiver questionnaires, direct observation of toothbrushing routines, and broader assessments of activities of daily living. Assessments targeted sensory issues such as taste sensitivity and bristle intolerance, motor challenges such as spitting and rinsing, and related oral habits such as mouthing or biting objects [20].

Toothbrushing difficulties were identified through parental reports of problems such as changing new toothbrushes, taste sensitivity (often manifesting as sampling of numerous brands before finding a suitable one), inability to spit water and toothbrushing intolerance. [20]

##### 3.3.2.2. Environmental Modifications

Environmental adaptations have been widely used to enhance accessibility, comfort and engagement in oral health care. Junnarkar et al. [20] and Grönbeck Linden et al. [33] reported recommendations such as adjusting the heights of sinks and mirrors, enabling seated brushing positions, and relocating brushing activities outside of bathrooms to reduce sensory overload. For older adults, access to mirrors and stable seating was prioritised [33]. In dental settings, modifications included noise reduction, encouragement, calming lighting and the provision of quiet waiting areas for individuals with sensory sensitivities [20].

This included exploring alternative locations or adjusting the bathroom environment, for example, fixing the sink and mirror at appropriate heights, or teaching spitting using coloured water to facilitate visual feedback… [20]

##### 3.3.2.3. Educational Strategies

Educational interventions targeted both clients and caregivers. Strategies included behavioural modelling, play‐based education, visual routine schedules and desensitisation [20]. For children, puppets, toys, models, social stories and preparatory videos were used to familiarise them with dental environments [20].

Behavioural strategies, such as parental or peer modelling, pretend play, using a visual schedule, desensitisation and structuring by counting or using a time‐based strategy (e.g., a timer or a song), were also thought to be useful in teaching toothbrushing and spitting. [20]

##### 3.3.2.4. Sensory‐Based Approaches

Sensory processing approaches were employed to prepare individuals for oral hygiene and to reduce aversion [16]. These included oral massage, facial stimulation, deep pressure, body brushing and jaw compression. Adapted tools, such as electric toothbrushes, finger brushes, and alternative toothbrush designs, were recommended to accommodate sensory and motor needs. Caregivers were trained to apply these interventions consistently as part of the daily routine [20].

…coping strategies such as wearing a weighted vest and having fidget toys are known OT interventions considered as ‘deep pressure’ techniques or ‘heavy work’ activities that are used to treat sensory defensiveness. [20]

#### 3.3.3. Perceived Enablers for OTs in Providing Oral Health Care

All included studies [19, 20, 33] reported a range of factors that supported OTs in delivering effective oral health care. These facilitators were grouped into five domains: understanding person‐related factors, knowledge and training, family‐centred practice, multidisciplinary team involvement and access to sufficient tools and resources.

##### 3.3.3.1. Understanding Person‐Related Factors

Understanding person‐related factors was a central facilitator across all studies, particularly in tailoring oral health care strategies to meet individual client needs. Junnarkar et al. [20] highlighted how knowledge of sensory sensitivities, such as aversions to specific textures or sounds, allowed therapists to suggest tailored coping strategies that made toothbrushing more tolerable. Therapists considered motor impairments when recommending appropriate oral health care tools or modifying tasks. In older populations, Grönbeck Linden et al. [33] noted that therapists accounted for cognitive changes, such as reduced memory or spatial awareness, to support effective oral health care routines. Emotional responses such as fear, discomfort or low motivation were also recognised as key components influencing engagement in oral hygiene, and therapists used graded support to address these needs [33].

Understanding the overall and oral sensory profile of the child was perceived as essential for suggesting coping strategies for toothbrushing, as was adjusting the environment to enable visual feedback. [20]

##### 3.3.3.2. Knowledge and Training

Knowledge and professional preparation were consistently described as important facilitators. Junnarkar et al. [20] reported that all OTs in their study had received training in sensory integration therapy, with some pursuing additional certifications relevant to paediatric care. This foundational knowledge enabled therapists to assess and manage complex oral health care challenges in children with ASD. The study also emphasised the importance of interprofessional education delivered by dental professionals to increase an OT’s ability to identify early oral health issues and make timely referrals [20]. Similarly, Curtin et al. [19] noted that although formal oral health care training was limited, OTs with greater awareness felt more confident integrating oral health into their routine care.

OTs and STs [speech therapists] are needed to explore the potential use of these tools and techniques in dentistry for persons with sensory processing and oral motor issues, particularly in applications to enable children to learn to spit, and facilitation of toothbrushing and dental treatment. [20]

##### 3.3.3.3. Family‐Centred Practice

All three studies acknowledged the importance of including family members and caregivers in the therapeutic process [19, 20, 33]. Junnarkar et al. [20] emphasised that caregivers, such as grandparents or domestic helpers, who often support daily routines, needed to be involved in consultation sessions to ensure that oral health care strategies were appropriately implemented at home. Including caregivers not only improved continuity of care but also enhanced the accuracy of OT assessments. This approach was particularly valuable in dual‐income households, where parents might not always be present during therapy sessions [20].

[it would] be advantageous for their [family’s] involvement in consultation sessions to facilitate clinicians’ understanding of the child’s sensory needs and challenges. [20]

##### 3.3.3.4. Multidisciplinary Support

Facilitators at the systems level included collaborative practice within multidisciplinary teams. Junnarkar et al. [20] described how coordinated support among OTs, STs and dental professionals enabled the development of individualised care, as noted in the quote below:

For successful dental visits, support from an interdisciplinary team, which considers their specific needs and the creation of an individualised plan, is ideally required. [20]

These interdisciplinary efforts were especially crucial for supporting children with ASD during dental visits, which are often anxiety‐inducing [20].

##### 3.3.3.5. Resources and Tools

The availability of appropriate tools and resources was identified as an important practical facilitator. Curtin et al. [19] reported that access to oral health care supplies supported OTs in delivering interventions:

Six participants mentioned how they felt that the current oral health assessment tool in use on the ward was helpful in the delivery of oral health care to patients. [19]

Additionally, Junnarkar et al. [20] encouraged therapists to explore tools from dentistry, including finger brushes, weighted vests, or alternative toothbrush designs, to address specific sensory or motor difficulties that limited participation in oral health care.

#### 3.3.4. Perceived Barriers for OTs in Providing Oral Health Care Delivery

All included studies identified several challenges that hindered OTs in their delivery of effective oral health care [19, 20, 33]. These barriers were categorised into five main domains: limited oral health literacy, person‐related challenges, insufficient knowledge of oral disease, systemic barriers and resource limitations.

##### 3.3.4.1. Limited Oral Health Literacy

Limited oral health literacy was reported in two studies as an important barrier to effective oral health care delivery. Junnarkar et al. [20] found that toothbrushing was often not prioritised by parents or caregivers, particularly in cases where the main caregiver was absent during therapy consultations. This lack of prioritisation meant that important oral health concerns were often overlooked or addressed only sporadically. In some cases, caregivers expressed confusion about the purpose and importance of fluoride toothpaste in preventing dental caries, leading to avoidance of its use altogether. Curtin et al. [19] similarly observed that in hospital rehabilitation settings, oral health care was often undervalued compared to other hygiene practices and rehabilitation priorities, resulting in reduced attention from staff.

Toothbrushing was, however, either not perceived to be a prime parental concern or not raised because the child’s main caregiver was not present at the visit. [20]

##### 3.3.4.2. Person‐Related Challenges

All three studies identified a range of person‐related challenges that hindered participation in oral hygiene routines. Junnarkar et al. [20] described sensory sensitivities such as aversion to toothpaste texture or flavour, intolerance to toothbrush bristles and distress caused by environmental triggers such as loud toilet flushes or water sounds. These factors often led to refusal or incomplete oral health care. Grönbeck Linden et al. [33] reported that older adults experienced physical limitations such as reduced upper body control, poor balance and difficulty leaning forward to spit, all of which affected task completion. Emotional and psychological factors, including the fear of pain, disgust at oral debris or bleeding gums and sadness, further discouraged participation. Cognitive deficits, such as memory loss and reduced spatial awareness, were noted to interfere with an individual’s ability to initiate, plan and complete oral hygiene tasks [2, 3].

Unique challenges of patient and stroke‐related factors. Patient‐related factors were mentioned as barriers to providing oral health care and advice to this patient group. [19]

##### 3.3.4.3. Insufficient Oral Disease Knowledge

Two studies reported that gaps in OT knowledge posed a barrier to effective oral health care delivery. Junnarkar et al. [20] found that therapists were uncertain about which toothpaste formulations were most appropriate for individuals with sensory sensitivities, including optimal fluoride levels for caries prevention. Some expressed uncertainty in advising parents about safe and effective oral health care strategies, particularly when children swallowed toothpaste. Curtin et al. [19] highlighted a lack of formal training among therapists in providing oral health care for individuals with dysphagia post‐stroke, leading to low confidence and uncertainty in addressing oral health needs within this population.

Participants in these focus groups overwhelmingly felt that a lack of knowledge about oral care for this population led to feelings of uncertainty and cautiousness when providing oral care or giving oral hygiene advice. [19]

##### 3.3.4.4. Systemic Barriers

System‐level issues further limited the delivery of effective oral health care. Junnarkar et al. [20] identified the absence of a clear and structured dental referral pathway, meaning that OTs often advised parents to locate a dentist independently, which sometimes delayed access to care. Curtin et al. [19] reported that within stroke rehabilitation units, oral health care was frequently deprioritised due to competing medical and therapy demands. The lack of specific guidelines for implementing oral health care interventions in this setting added to the challenge as OTs were unsure of the best practices to follow. Limited access to dental professionals within hospital teams compounded these difficulties [19].

The guidelines do not give specific advice or direction for members of the stroke team on how to provide this care or what specific interventions to implement. [19]

##### 3.3.4.5. Resource Limitations

Resource constraints were also identified as a barrier to delivering optimal oral health care. Curtin et al. [19] reported that OTs often lacked access to essential oral health care supplies, including mirrors, denture fixatives and suction toothbrushes. These limitations restricted their ability to provide thorough and tailored interventions. Junnarkar et al. [20] noted that the absence of adequate preparation resources, such as sensory‐friendly dental tools or pre‐visit acclimatisation aids, limited the effectiveness of pre‐dental visit training for children with ASD.

Difficulties accessing key items such as mirrors, denture fixatives and suction toothbrushes affect the delivery of oral care. [19]

### 3.4. Assessment of Methodological Quality

The methodological quality of the three included studies, assessed using the JBI Critical Appraisal Checklist for Qualitative Research, was generally high, with all studies meeting at least 70% of the checklist items (Table S4). Grönbeck Linden et al. [33] met 7 out of 10 items, with limitations including a lack of explicit philosophical positioning and an absence of reflexivity; one item was rated as unclear. Curtin et al. [19] met 8 out of 10 items; the unclear items related to the philosophical perspective and researcher positioning. Junnarkar et al. [20] also met 8 out of 10 items as they did not address researcher reflexivity or cultural/theoretical positioning. Common methodological gaps across studies included limited reporting on the philosophical underpinnings and insufficient discussion of the potential influence of researchers on the research process, which may limit study methodological transparency and compromise the overall trustworthiness of qualitative synthesis. Despite these limitations, all studies demonstrated strong alignment between research aims, methodology, data collection and analysis, with clear representation of participants’ perspectives and evidence of ethical approval, supporting their inclusion in the synthesis [19, 20, 33].

### 3.5. Confidence in the Review Findings

The GRADE‐CERQual approach was used to assess confidence in each of the four review findings, based on methodological limitations, coherence, adequacy of data and relevance (Table S5).

Confidence in the finding relating to OTs’ knowledge of oral health care was assessed as moderate. Although the contributing studies [19, 20, 33] were methodologically sound, minor coherence concerns arose from the apparent difference in the depth of OT knowledge reported between settings. OTs in the acute stroke unit [19] described limited oral health knowledge, whereas OTs working with children with ASD [20] demonstrated more specific knowledge of sensory‐related oral behaviours and fluoride use. This variation was interpreted as reflecting differences in population and practice context rather than a genuine contradiction, but it nonetheless tempered confidence, as did the relatively narrow evidence base of only three primary studies spanning markedly different clinical populations.

The finding describing current OT practices (assessment, environmental modification, education and sensory‐based approaches) was also assessed as moderate confidence. This finding drew on only two of the three included studies [20, 33], as Curtin et al. [19] did not describe a defined practice repertoire among stroke unit OTs, which reduced both adequacy and relevance, particularly with respect to the transferability of these practices to acute inpatient settings.

Confidence in the finding relating to perceived enablers was assessed as moderate to high. All three studies contributed data that were consistent in direction, and the finding was well supported by illustrative data. However, two subthemes, that is, multidisciplinary support and family‐centred practice, relied predominantly on a single study [20], which limited the extent to which these specific enablers could be generalised across settings.

The finding relating to perceived barriers was assessed as high confidence, reflecting strong coherence across all three studies, richly supported data across nearly every subtheme, and only minor relevance concerns relating to the setting‐specific nature of some systemic and resource barriers.

Overall, confidence in the review findings ranged from moderate to high. The main factor limiting confidence across findings was the small number of primary studies (*n* = 3), which constrained the adequacy and relevance domains.

## 4. Discussion

This review aimed to identify the current knowledge and practices of OTs in oral health, as well as the key facilitators and barriers influencing their role in promoting oral health within their scope of practice. Three qualitative studies met the inclusion criteria [19, 20, 33]. These studies were conducted across diverse settings, including paediatric, neurorehabilitation and aged care settings, and involved OTs working either independently or as part of multidisciplinary teams. By adopting varied qualitative methods, these studies gathered perspectives from practitioners on their understanding of oral health, their practical interventions, and the organisational and systemic factors influencing service delivery [19, 20, 33].

Although the eligibility criteria of this review included quantitative, qualitative and mixed‐methods studies, only three qualitative studies met the inclusion criteria. This suggests limited existence of evidence on the role of OTs in oral health promotion and that the available literature is mostly exploratory in nature. Furthermore, the availability of only qualitative evidence suggests that researchers are interested in understanding the experiences, perceptions and facilitators and barriers related to oral health care practices.

The findings of this review indicate that OTs possess valuable knowledge in recognising oral health conditions, understanding factors influencing health outcomes and addressing the complexity of oral hygiene tasks [19, 20, 33]. Their practices often involve activity analysis, environmental modification, education and sensory‐based strategies to promote autonomy and enable participation in daily oral health care routines. Across all included studies, facilitators to this work included interventions tailored by a comprehensive understanding of the person, integration of family‐centred approaches, multidisciplinary collaboration, targeted training and access to adequate resources. Barriers included limited oral health literacy, person‐related challenges, clear gaps in therapist knowledge and confidence, systemic obstacles such as unclear referral pathways and competing priorities, and resource constraints [19, 20, 33].

The following sections synthesise findings directly supported by the included studies (Sections 4.1–4.4). Section 4.5 offers an interpretive framework linking these findings, proposed by the authors, while Sections 4.6 and 4.7 discuss the review’s limitations and implications that extend beyond what the evidence directly supports.

### 4.1. OT Knowledge on Oral Health Care

The included studies demonstrated that OTs can recognise oral health conditions, understand personal and environmental factors influencing oral health outcomes and address the complexity of oral hygiene tasks [19, 20, 33]. This knowledge reflects the profession’s core skill in activity analysis, enabling therapists to identify the multifaceted motor, sensory, cognitive, emotional and environmental factors of oral health care. Sensory sensitivities, such as aversion to toothpaste texture or toothbrush bristles, were understood as barriers to participation, while motor limitations like apraxia or hypotonia were recognised as impairments requiring adaptive strategies [19, 20, 33]. These findings align with previous literature, highlighting the unique perspective OTs bring to preventive oral health care [34–37]. However, the studies also revealed variability in the depth of oral health knowledge across practice contexts, particularly regarding specialist needs such as dysphagia management [38] or fluoride recommendations [39]. According to a study of OT students, 78.1% did not receive any formal oral health education during their degree programme, and 31.9% were not aware that working with dental professionals is part of their scope of practice [40]. As a result, many OTs are unsure of which oral conditions, like periodontal disease or dental caries, they are assisting in preventing through hygiene measures. Broader evidence supports the need for targeted oral health education for non‐dental health professionals to address these gaps [41–43]. A study by de Sam Lazaro et al. [44] proposed oral health competency‐based education, such as asynchronous webinars, as an effective approach to enhance self‐reported oral health knowledge among allied health students. Likewise, Gill et al. [45] proposed several approaches to the integration of oral health education into health professional school curriculums, such as through interprofessional education, clinical experience, online educational modules and case‐based learning.

Notably, the literature mostly focuses on identifying barriers rather than the implementation of structured oral health interventions or collaborative strategies by OTs. This limits translation into practice and indicates gaps in interdisciplinary collaboration and professional training.

### 4.2. OT Practices in Providing Oral Health Care

The review found that OTs contribute to oral health care primarily through assessments, environmental modifications, education and sensory strategies. OT assessments captured sensory, motor, cognitive and behavioural factors affecting oral health care, often incorporating caregiver input to ensure interventions were contextually relevant [20]. Environmental modifications such as adapted bathroom layouts, appropriate seating, and mirror positioning were applied to maximise independence and safety, particularly in older adults or those with physical impairments [33]. Educational strategies included modelling, visual schedules, desensitisation techniques and caregiver training, while sensory approaches such as oral massage, deep pressure techniques and gradual exposure were used to increase tolerance for oral health care routines [19, 20, 33]. These practices appear consistent with the preventive and enabling focus of occupational therapy and may complement dental care by promoting autonomy and integration of oral hygiene into daily routines [18, 46].

While these interventions were reported as helpful within their respective settings, the review also noted that occupational therapy practices in oral health care are inconsistently implemented across different health care contexts. OTs work across diverse settings, for example, adapting bathroom environments in a hospital or school setting or in a clinic. They also work with a wide range of populations, including children, older adults and people with stroke. Addressing oral health care lies within the broader self‐care routines of these participants. This variability may be shaped by systemic factors, such as competing priorities within rehabilitation and limited time allocations for oral health promotion [20, 33]. Therefore, oral health care intervention can be context‐specific or population‐tailored. These findings are consistent with previous studies, which similarly identified that while OTs can effectively integrate oral hygiene into functional routines, uptake is often hindered by training gaps, inconsistent institutional protocols and limited interdisciplinary coordination [34, 35].

### 4.3. Enablers for OT’s Involvement in Oral Health Care

Key facilitators identified across the studies included a strong understanding of factors related to the person, family‐centred care, multidisciplinary collaboration, existing training and access to resources [19, 20, 33]. These findings are consistent with the Health Resources and Service Administration recommendations on the core competencies required for non‐dental health professions involved in promotion of oral health. These competencies include preventive interventions, oral health risk assessment, interprofessional collaboration and patient education [47, 48]. An OT’s ability to individualise interventions, accounting for sensory profiles, motor skills, cognitive status and emotional readiness, was reported as a key perceived enabler [18, 20]. Enabling family engagement, by methods such as involving caregivers in training sessions, was particularly important in sustaining oral hygiene routines at home [49]. Training in sensory integration, especially in paediatric settings, provided OTs with essential skills to manage complex oral health care needs [20]. Interprofessional education delivered by dental professionals further enhanced OTs’ abilities to identify early oral health issues and make timely referrals [19, 41]. Multidisciplinary collaboration, especially in cases involving children with ASD or adults who were post‐stroke, enabled comprehensive, individualised care planning [19, 35, 50]. Access to appropriate tools and resources, such as toothbrushes, adaptive aids and oral health assessment instruments, facilitated timely intervention [19, 35].

### 4.4. Barriers for OT Involvement in Oral Health Care

The review identified multiple barriers, including limited oral health literacy among both OTs and caregivers, person‐related challenges, insufficient knowledge, systemic obstacles and resource constraints [19, 20, 33]. In many cases, oral health care is undervalued within rehabilitation, leading to its deprioritisation in daily routines. Caregiver misconceptions, such as uncertainty about fluoride use, further limited adherence. In addition, researchers found person‐related challenges that hindered participation, including resistance, pain, sensory aversions, motor deficits and cognitive decline [19, 20, 33]. Gaps in OT knowledge, particularly regarding specialist oral health care needs, also restricted effective intervention.

Systemic barriers such as unclear referral pathways, basic oral disease knowledge, absence of formal guidelines, competing clinical priorities and limited interdisciplinary integration were consistently reported [19, 20, 33]. These organisational barriers are not unique to occupational therapy. Reviews of other health professions have suggested limited time, lack of material resources, inadequate oral health training, challenges to accessing dental care and insufficient funding as the major organisational level barriers involved in prevention and promotion of oral health care [34, 51]. Findings from other studies also suggest that, in a multidisciplinary environment, unclear responsibilities regarding oral health care can complicate interprofessional relationships, and referral practices are often absent [52]. Awareness of the necessity of oral health care among health care professionals should be encouraged [52]. Finally, inadequate access to oral health care supplies and adaptive equipment constrains the scope of services [19, 20, 33]. These findings mirror challenges observed in integrating other preventive health measures into allied health practice [34, 53, 54].

### 4.5. Interrelationship Between Knowledge, Practice, Facilitators and Barriers

The following section offers an interpretive synthesis, proposed by the authors, of how the identified knowledge, practices, facilitators and barriers may relate to one another and should be regarded as a conceptual framework for future investigation.

The interaction between what OTs know, what they do in practice, and the barriers and facilitators they encounter is complex. Foundational knowledge appears to enable therapists to design and deliver tailored interventions; for example, adaptive strategies enable OTs to integrate oral health care into self‐care routines. However, knowledge alone does not guarantee implementation. The extent to which these tailored interventions translate into practice depends on the availability of training, resources and organisational support [19, 20, 33, 34]. Family‐centred care and multidisciplinary collaboration can help address person‐related challenges [49, 50]. Similarly, targeted training and clear clinical guidelines can mitigate uncertainty in practice by providing OTs with structured protocols and the confidence to act [38, 41, 43]. Importantly, these facilitators may plausibly counter key barriers, although this interaction was not directly examined within the included studies: interprofessional education addresses knowledge gaps, organisational endorsement reduces role ambiguity and resource toolkits overcome practical constraints. Yet, without systemic changes, integrating oral health into policy frameworks, allocating time and staffing and maintaining quality standards may have a limited impact [34, 36]. This suggests that integration of oral health care into occupational therapy practice requires a multi‐level approach: strengthening individual competencies, fostering collaborative practice, and ensuring institutional and policy‐level support to sustain implementation.

### 4.6. Strengths and Limitations

To the best of our knowledge, this is the first systematic review to synthesise preliminary evidence on OT knowledge and practices in oral health care, as well as the barriers and facilitators influencing the integration of oral health care into occupational therapy practice. This review offers a synthesis of how OTs approach oral health care, providing insights into both the profession’s potential and the current limitations of its role. It highlights occupational therapy’s person‐centred approach, adaptability across diverse populations and settings, and the shared challenges faced when integrating oral health care into routine practice. Key strengths include the use of an exhaustive search strategy across multiple databases without restrictions on publication date or region, citation chasing of relevant literature, and rigorous quality appraisal using the JBI Critical Appraisal Checklist for Qualitative Research [31]. The findings are further strengthened by their grounding in methodologically robust qualitative studies, which offer rich contextual detail.

However, several limitations must be acknowledged. Only three qualitative studies met the eligibility criteria, limiting the strength of conclusions that can be drawn and warranting explicit consideration when interpreting the findings. While the small number of eligible studies may genuinely reflect a paucity of qualitative research examining the perspectives of OTs in relation to oral health care, it nonetheless means that each of the four review findings is derived from a maximum of three and, in some cases, only two primary studies. As reflected in the GRADE‐CERQual assessment, this limited evidence base constrained the adequacy of data underpinning several findings, particularly those relating to current OT practices and specific enablers such as multidisciplinary support. The certainty and transferability of these findings should be considered. Certainty, as captured by the GRADE‐CERQual ratings, reflects whether the studies that exist agree with one another and provide sufficiently rich data. Transferability, by contrast, concerns whether these findings are likely to apply beyond the specific populations and settings studied. This distinction is particularly important here, as the three included studies drew on markedly different clinical populations and contexts: acute stroke inpatients [19], community‐dwelling older adults [33] and children with ASD [20]. While the recurrence of similar barriers (e.g., limited oral health training, absence of clear referral pathways and competing clinical priorities) across such divergent populations lends some support to the plausibility of these findings more broadly, it cannot be assumed that they generalise to other populations relevant to OT practice, such as adults with intellectual disability, palliative care patients, or individuals with acquired brain injury outside the acute stroke context. Similarly, findings relating to specific practices and enablers should be interpreted as context‐specific illustrations of what is possible rather than as evidence of what is typical or effective across OT practice more broadly. Future research conducted across a wider range of clinical populations and practice settings is needed to test whether the barriers, enablers and practices identified here hold more broadly and to build a more certain evidence base from which practice recommendations and training curricula can be more confidently developed.

To avoid framing all review findings within the occupational therapy practice, it is noteworthy that some evidence derived from included studies reflects broader perspectives of the multidisciplinary team, and OTs were not the primary participants. The small and specific participant pools and qualitative designs may limit broader applicability, and methodological diversity across studies made direct comparison challenging. The exclusive focus on qualitative research introduces subjectivity that may limit replicability, and the absence of quantitative data prevents assessment of the scale or statistical significance of the observed patterns. Additionally, restricting inclusion to peer‐reviewed, English‐language publications may have excluded relevant studies, and outcome reporting bias remains a potential risk.

Although OTseeker was listed as a database to be searched in the review protocol, it was not included in the final search strategy primarily because our initial search showed that articles from OTseeker substantially overlap with the content of other databases (e.g., MEDLINE) included in the study. Therefore, this is a deviation from the protocol, and it is possible that records were not captured as a result.

### 4.7. Implications for Practice and Research

This review suggests that OTs may be well‐positioned to support oral health care for individuals who have a limited ability to perform oral hygiene independently. However, considering the limited evidence available from qualitative studies, this potential should be cautiously interpreted. The findings suggest that targeted strategies can be used to address key identified barriers. Integrating oral health training into occupational therapy curriculum and offering ongoing professional development may directly tackle perceived knowledge gaps and build confidence in the practice. Establishing clear referral pathways to dental professionals may mitigate systemic obstacles and strengthen continuity of care, while integrating oral health into standard occupational therapy assessment and intervention protocols and aligning with standard clinical guidelines may help reduce role ambiguity and uncertainty. The allocation of resources, as well as access to adaptive tools and culturally appropriate educational materials, may further enable OTs to tailor interventions for diverse populations, and enhancing multidisciplinary collaboration, including partnerships with community‐based dental services, may promote access and sustainability. This review relied on preliminary findings from limited qualitative evidence; future research should incorporate mixed‐methods and quantitative designs to measure the effectiveness of OT‐led oral health interventions, including relevant quantitative oral health outcomes, such as plaque scores, incidence of oral disease, aspiration pneumonia, functional independence in oral hygiene, caregiver adherence and referral rates. Linking these outcomes with qualitative insights would provide a comprehensive understanding of impact and implementation, informing systemic and educational improvements that would embed oral health within routine occupational therapy practice.

## 5. Conclusion

OTs possess valuable knowledge and skills that may be applied to oral health care and could contribute to preventive care and support individuals in maintaining oral hygiene as part of their daily lives. The evidence derived from the limited number of studies included in this review suggests preliminary insights to current occupational therapy practice which is constrained by barriers at individual, organisational and systemic levels. Addressing these barriers through targeted education, adequate resourcing and stronger interprofessional collaboration may support more active involvement of OTs in promoting oral health and reducing preventable oral health problems across diverse populations. However, further research is required to better understand the feasibility and effectiveness of these approaches among different populations and settings.

## Funding

This study received no specific funding.

## Disclosure

All authors have read and approved the final version of the manuscript. The corresponding author had full access to all data in this study and takes complete responsibility for the integrity of the data and the accuracy of the data analysis.

## Conflicts of Interest

The authors declare no conflicts of interest.

## Supporting Information

Additional supporting information can be found online in the Supporting Information section.

## Supporting information


**Supporting Information** Table S1: PRISMA checklist. Table S2: Search strategies. Table S3: Exclusion reasons. Table S4: JBI quality assessment of included studies. Table S5: GRADE‐CERQual summary of qualitative findings.

## Data Availability

The authors confirm that the data supporting the findings of this study are available within the article and its Supporting Information.
