# Supplementary material for: Integrating Oral Health Into Occupational Therapy: Knowledge, Practices, Barriers and Facilitators—A Systematic Review
Source: Int J Dent. 2026 Oct 12;2026:2007891. doi: 10.1155/ijod/2007891 (PMC13656200; doi:10.1155/ijod/2007891)
Supplement: Supplementary file 1 [file IJOD-2026-2007891-s001.docx]

# **Supplementary Table 1.** PRISMA Checklist

| **Section and Topic** | **Item #** | **Checklist item** | **Location where item is reported** |
| --- | --- | --- | --- |
| **TITLE** | | |  |
| Title | 1 | Identify the report as a systematic review. | 1 |
| **ABSTRACT** | | |  |
| Abstract | 2 | See the PRISMA 2020 for Abstracts checklist. | 2-3 |
| **INTRODUCTION** | | |  |
| Rationale | 3 | Describe the rationale for the review in the context of existing knowledge. | 4-6 |
| Objectives | 4 | Provide an explicit statement of the objective(s) or question(s) the review addresses. | 4-6 |
| **METHODS** | | |  |
| Eligibility criteria | 5 | Specify the inclusion and exclusion criteria for the review and how studies were grouped for the syntheses. | 7 |
| Information sources | 6 | Specify all databases, registers, websites, organisations, reference lists and other sources searched or consulted to identify studies. Specify the date when each source was last searched or consulted. | 7 |
| Search strategy | 7 | Present the full search strategies for all databases, registers and websites, including any filters and limits used. | 7-8 |
| Selection process | 8 | Specify the methods used to decide whether a study met the inclusion criteria of the review, including how many reviewers screened each record and each report retrieved, whether they worked independently, and if applicable, details of automation tools used in the process. | 8 |
| Data collection process | 9 | Specify the methods used to collect data from reports, including how many reviewers collected data from each report, whether they worked independently, any processes for obtaining or confirming data from study investigators, and if applicable, details of automation tools used in the process. | 8 |
| Data items | 10a | List and define all outcomes for which data were sought. Specify whether all results that were compatible with each outcome domain in each study were sought (e.g. for all measures, time points, analyses), and if not, the methods used to decide which results to collect. | 9 |
|  | 10b | List and define all other variables for which data were sought (e.g. participant and intervention characteristics, funding sources). Describe any assumptions made about any missing or unclear information. | 9 |
| Study risk of bias assessment | 11 | Specify the methods used to assess risk of bias in the included studies, including details of the tool(s) used, how many reviewers assessed each study and whether they worked independently, and if applicable, details of automation tools used in the process. | 10 |
| Effect measures | 12 | Specify for each outcome the effect measure(s) (e.g. risk ratio, mean difference) used in the synthesis or presentation of results. | N/A |
| Synthesis methods | 13a | Describe the processes used to decide which studies were eligible for each synthesis (e.g. tabulating the study intervention characteristics and comparing against the planned groups for each synthesis (item #5)). | 9 |
|  | 13b | Describe any methods required to prepare the data for presentation or synthesis, such as handling of missing summary statistics, or data conversions. | 9 |
|  | 13c | Describe any methods used to tabulate or visually display results of individual studies and syntheses. | N/A |
|  | 13d | Describe any methods used to synthesize results and provide a rationale for the choice(s). If meta-analysis was performed, describe the model(s), method(s) to identify the presence and extent of statistical heterogeneity, and software package(s) used. | N/A |
|  | 13e | Describe any methods used to explore possible causes of heterogeneity among study results (e.g. subgroup analysis, meta-regression). | N/A |
|  | 13f | Describe any sensitivity analyses conducted to assess robustness of the synthesized results. | N/A |
| Reporting bias assessment | 14 | Describe any methods used to assess risk of bias due to missing results in a synthesis (arising from reporting biases). | 10 |
| Certainty assessment | 15 | Describe any methods used to assess certainty (or confidence) in the body of evidence for an outcome. | N/A |
| **RESULTS** | | |  |
| Study selection | 16a | Describe the results of the search and selection process, from the number of records identified in the search to the number of studies included in the review, ideally using a flow diagram. | 11-13 |
|  | 16b | Cite studies that might appear to meet the inclusion criteria, but which were excluded, and explain why they were excluded. | 11-13 |
| Study characteristics | 17 | Cite each included study and present its characteristics. | 13-18 |
| Risk of bias in studies | 18 | Present assessments of risk of bias for each included study. | 27-29 |
| Results of individual studies | 19 | For all outcomes, present, for each study: (a) summary statistics for each group (where appropriate) and (b) an effect estimate and its precision (e.g. confidence/credible interval), ideally using structured tables or plots. | 28-29 |
| Results of syntheses | 20a | For each synthesis, briefly summarise the characteristics and risk of bias among contributing studies. | 27-19 |
|  | 20b | Present results of all statistical syntheses conducted. If meta-analysis was done, present for each the summary estimate and its precision (e.g. confidence/credible interval) and measures of statistical heterogeneity. If comparing groups, describe the direction of the effect. | N/A |
|  | 20c | Present results of all investigations of possible causes of heterogeneity among study results. | N/A |
|  | 20d | Present results of all sensitivity analyses conducted to assess the robustness of the synthesized results. | N/A |
| Reporting biases | 21 | Present assessments of risk of bias due to missing results (arising from reporting biases) for each synthesis assessed. | N/A |
| Certainty of evidence | 22 | Present assessments of certainty (or confidence) in the body of evidence for each outcome assessed. | N/A |
| **DISCUSSION** | | |  |
| Discussion | 23a | Provide a general interpretation of the results in the context of other evidence. | 29-39 |
|  | 23b | Discuss any limitations of the evidence included in the review. | 36-38 |
|  | 23c | Discuss any limitations of the review processes used. | 36-38 |
|  | 23d | Discuss implications of the results for practice, policy, and future research. | 38 |
| **OTHER INFORMATION** | | |  |
| Registration and protocol | 24a | Provide registration information for the review, including register name and registration number, or state that the review was not registered. | 6 |
|  | 24b | Indicate where the review protocol can be accessed, or state that a protocol was not prepared. | 6 |
|  | 24c | Describe and explain any amendments to information provided at registration or in the protocol. | n/a |
| Support | 25 | Describe sources of financial or non-financial support for the review, and the role of the funders or sponsors in the review. | 39 |
| Competing interests | 26 | Declare any competing interests of review authors. | 39 |
| Availability of data, code and other materials | 27 | Report which of the following are publicly available and where they can be found: template data collection forms; data extracted from included studies; data used for all analyses; analytic code; any other materials used in the review. | 39 |

## **Supplementary Table 2. Search strategies**

**Ovid MEDLINE(R) ALL**

1. exp Oral Health/ or exp Oral Hygiene/ or exp Dental Care/ or exp Dental Care for Children/ or exp Dental Care for Disabled/ or exp Gingivitis/ or exp Periodontal Diseases/ or exp Chronic Periodontitis/ or exp Dental Plaque/ or exp Periodontitis/ or exp Tooth Diseases/ or exp Dental Caries/ or exp Mouth Diseases/ or exp Mouth Neoplasms/ or exp Tooth Injuries/ or exp Dentition, Mixed/ or exp Dentition/ or exp Dentition, Permanent/ or exp Toothache/ or exp Toothbrushing/ or exp Education, Dental/ or Dental Care for Aged/ or Denture Stomatitis/

2. (oral health* or dental health* or oral disease* or dental disease* or dental caries* or tooth decay* or oral trauma* or gum disease* or periodont* or gingiv* or oral hygiene* or mouth diseas* or dental car* or mouth tumo?r* or Mouth Neoplasm* or mouth cancer* or Oral cancer* or Oral traum* or dental visit* or tooth loss* or dental status* or dental pain* or dentition* or teeth* or tooth diseas* or dental care* or Tooth Injur* or toothache* or toothbrushing* or dental educat* or teeth diseas* or denture clean* or Dental Plaque* or tooth Plaque* or Denture Stomatitis or mouth hygiene or tooth care).mp.

3. ((Oral or dental or gum) adj2 (health* or hygiene* or car* or treatment* or visit* or diseas* or plaque)).tw,kw.

4. 1 or 2 or 3

5. Occupational Therapy/ or Occupational Therapists/ or patient care team/ or "activities of daily living"/

6. (occupational therap* or ergotherap* or OT therap* or "activities of daily living").mp.	116591

7. (Allied health* or Multidisciplinary team* or Multi-disciplinary team* or patient care team*).tw,kw.

8. 5 or 6 or 7

9. 4 and 8

10. Health Knowledge, Attitudes, Practice/ or Health behavior/ or Attitude to health/ or health knowledge, attitudes, practice/ or capacity building/ or health communication/ or "attitude of health personnel"/

11. (Health behavio?r or "Attitude to health" or health belief* or health practice* or health literac* or knowledge or attitude* or practice* or procedure* or interdisciplinary or multidisciplinary or interprofessional*).mp.

12. (barrier* or impediment* or challenge* or hindrance* or obstacle* or hurdle* or obstruction* or deterrent* or facilitator*).mp.

13. 10 or 11 or 12

14. 9 and 13

**Scopus**

( TITLE-ABS-KEY ( "oral health*" OR "dental health*" OR "oral disease*" OR "dental disease*" OR "dental caries*" OR "tooth decay*" OR "oral trauma*" OR "gum disease*" OR periodont* OR gingiv* OR "oral hygiene*" OR "mouth diseas*" OR "dental car*" OR "mouth tumo*r*" OR "Mouth Neoplasm*" OR "mouth cancer*" OR "Oral cancer*" OR "Oral traum*" OR "dental visit*" OR "tooth loss*" OR "dental status*" OR "dental pain*" OR dentition* OR teeth* OR "tooth diseas*" OR "dental care*" OR "Tooth Injur*" OR toothache* OR toothbrushing* OR "dental educat*" OR "teeth diseas*" OR "denture clean*" OR "Dental Plaque*" OR "Denture Stomatitis" OR mouth hygiene ) OR TITLE-ABS-KEY ( ( ( oral OR dental OR gum ) W/2 ( health* OR hygiene* OR car* OR treatment* OR visit* OR diseas* OR plaque ) ) ) AND TITLE-ABS-KEY ( "occupational therap*" OR ergotherap* OR "OT therap*" OR "activities of daily living" ) OR TITLE-ABS-KEY ( "Allied health*" OR "Multidisciplinary team*" OR "Multi-disciplinary team*" OR "patient care team*" ) AND TITLE-ABS-KEY ( "Health behavio*r" OR "Attitude to health" OR "health belief*" OR "health practice*" OR "health literac*" OR knowledge OR attitude* OR practice* OR procedure* OR interdisciplinary OR multidisciplinary OR interprofessional* ) OR TITLE-ABS-KEY ( ( barrier* OR impediment* OR challenge* OR hindrance* OR obstacle* OR hurdle* OR obstruction* OR deterrent* OR facilitator* ) ) )

**Embase Classic+Embase**

1	mouth hygiene/

2	gingivitis/

3	exp periodontal disease/

4	chronic periodontitis/

5	tooth plaque/

6	exp periodontitis/

7	exp tooth disease/

8	dental caries/

9	exp mouth disease/

10	mouth tumor/

11	tooth injury/

12	dentition/ or mixed dentition/ or primary dentition/ or secondary dentition/

13	tooth pain/ or tooth disease/

14	tooth brushing/

15	dental education/

16	exp dental procedure/

17	stomatitis/

18	(oral health* or dental health* or oral disease* or dental disease* or dental caries* or tooth decay* or oral trauma* or gum disease* or periodont* or gingiv* or oral hygiene* or mouth diseas* or dental car* or mouth tumo?r* or Mouth Neoplasm* or mouth cancer* or Oral cancer* or Oral traum* or dental visit* or tooth loss* or dental status* or dental pain* or dentition* oth diseas* or dental care* or Tooth Injur* or toothache* or toothbrushing* or dental educat* or denture clean* or Dental Plaque* or tooth Plaque* or Denture Stomatitis or mouth hygiene or tooth care).mp.

19	((Oral or dental or gum) adj2 (health* or hygiene* or car* or treatment* or visit* or diseas* or plaque)).tw,kw.

20	1 or 2 or 3 or 4 or 5 or 6 or 7 or 8 or 9 or 10 or 11 or 12 or 13 or 14 or 15 or 16 or 17 or 18 or 19

21	occupational therapy/ or occupational therapy assistant/

22	occupational therapist/

23	patient care team/

24	daily life activity/

25	(occupational therap* or ergotherap* or OT therap* or "activities of daily living").mp.

26	(Allied health* or Multidisciplinary team* or Multi-disciplinary team* or patient care team*).tw,kw.

27	21 or 22 or 23 or 24 or 25 or 26

28	attitude to health/

29	health behavior/

30	capacity building/

31	health personnel attitude/

32	(Health behavio?r or "Attitude to health" or health belief* or health practice* or health literac* or knowledge or attitude* or practice* or procedure* or interdisciplinary or multidisciplinary or interprofessional*).mp.

33	(barrier* or impediment* or challenge* or hindrance* or obstacle* or hurdle* or obstruction* or deterrent* or facilitator*).mp.

34	28 or 29 or 30 or 31 or 32 or 33

35	20 and 27 and 34

**CINAHL**

S35	S21 AND S27 AND S34	Expanders - Apply equivalent subjects	Interface - EBSCOhost Research Databases

S34	S28 OR S29 OR S30 OR S31 OR S32 OR S33	Expanders - Apply equivalent subjects	Interface - EBSCOhost Research Databases

S33	(barrier* OR impediment* OR challenge* OR hindrance* OR obstacle* OR hurdle* OR obstruction* OR deterrent* OR facilitator* )	Expanders - Apply equivalent subjects	Interface - EBSCOhost Research Databases

S32	("Health behavio#r" OR "Attitude to health" OR "health belief*" OR "health practice*" OR "health literac*" OR knowledge OR attitude* OR practice* OR procedure* OR interdisciplinary OR multidisciplinary OR interprofessional*)	Expanders - Apply equivalent subjects	Interface - EBSCOhost Research Databases

S31	(MH "Attitude of Health Personnel+")	Expanders - Apply equivalent subjects	Interface - EBSCOhost Research Databases

S30	(MH "Attitude to Health+")	Expanders - Apply equivalent subjects	Interface - EBSCOhost Research Databases

S29	(MH "Health Behavior+")	Expanders - Apply equivalent subjects	Interface - EBSCOhost Research Databases

S28	(MH "Attitude to Health+") OR (MH "Health Knowledge")	Expanders - Apply equivalent subjects	Interface - EBSCOhost Research Databases

S27	S22 OR S23 OR S24 OR S25 OR S26	Expanders - Apply equivalent subjects	Interface - EBSCOhost Research Databases

S26	"occupational therap*" OR ergotherap* OR "OT therap*" OR "activities of daily living" OR "Allied health*" OR "Multidisciplinary team*" OR "Multi-disciplinary team*" OR "patient care team*"	Expanders - Apply equivalent subjects	Interface - EBSCOhost Research Databases

S25	(MH "Activities of Daily Living+")	Expanders - Apply equivalent subjects	Interface - EBSCOhost Research Databases

S24	(MH "Multidisciplinary Care Team+")	Expanders - Apply equivalent subjects	Interface - EBSCOhost Research Databases

S23	(MH "Pediatric Occupational Therapists") OR (MH "Occupational Therapists+") OR (MH "Canadian Association of Occupational Therapists") OR (MH "British Association and College of Occupational Therapists")	Expanders - Apply equivalent subjects	Interface - EBSCOhost Research Databases

S22	(MH "Occupational Therapy+")	Expanders - Apply equivalent subjects	Interface - EBSCOhost Research Databases

S21	S1 OR S2 OR S3 OR S4 OR S5 OR S6 OR S7 OR S8 OR S9 OR S10 OR S11 OR S12 OR S13 OR S14 OR S15 OR S16 OR S17 OR S18 OR S19 OR S20	Expanders - Apply equivalent subjects	Interface - EBSCOhost Research Databases

S20	((Oral OR dental OR gum ) N2 (health* OR hygiene* OR car* OR treatment* OR visit* OR diseas* OR plaque ))	Expanders - Apply equivalent subjects	Interface - EBSCOhost Research Databases

S19	"oral health*" OR "dental health*" OR "oral disease*" OR "dental disease*" OR "dental caries*" OR "tooth decay*" OR "oral trauma*" OR "gum disease*" OR periodont* OR gingiv* OR "oral hygiene*" OR "mouth diseas*" OR "dental car*" OR "mouth tumo#r*" OR "Mouth Neoplasm*" OR "mouth cancer*" OR "Oral cancer*" OR "Oral traum*" OR "dental visit*" OR "tooth loss*" OR "dental status*" OR "dental pain*" OR dentition* OR teeth* OR "dental care*" OR "Tooth Injur*" OR toothache* OR toothbrushing* OR "tooth brushing" OR "dental educat*" OR "teeth diseas*" OR "denture clean*" OR "Dental Plaque*" OR "tooth Plaque*" OR "Denture Stomatitis" OR "mouth hygiene" OR "tooth care"	Expanders - Apply equivalent subjects	Interface - EBSCOhost Research Databases

S18	MH "Education, Dental") OR (MH "Education, Dental Hygiene") OR (MH "Dental Health Education")	Expanders - Apply equivalent subjects	Interface - EBSCOhost Research Databa

S17	(MH "Dentition+") OR (MH "Dentition, Mixed") OR (MH "Dentition, Primary") OR (MH "Dentition, Secondary")	Expanders - Apply equivalent subjects	Interface - EBSCOhost Research Databases

S16	(MH "Chronic Periodontitis")	Expanders - Apply equivalent subjects	Interface - EBSCOhost Research Databases

S15	MH "Gingivitis+") OR (MH "Gingival Diseases+") OR (MH "Gingival Neoplasms")	Expanders - Apply equivalent subjects	Interface - EBSCOhost Research Databases

S14	(MH "Stomatitis, Denture")	Expanders - Apply equivalent subjects	Interface - EBSCOhost Research Databases

S13	(MH "Education, Dental") OR (MH "Education, Dental Hygiene") OR (MH "Dental Health Education")	Expanders - Apply equivalent subjects	Interface - EBSCOhost Research Databases

S12	(MH "Toothbrushing")	Expanders - Apply equivalent subjects	Interface - EBSCOhost Research Databases

S11	(MH "Toothache")	Expanders - Apply equivalent subjects	Interface - EBSCOhost Research Databases

S10	(MH "Dentition+") OR (MH "Dentition, Mixed") OR (MH "Dentition, Primary") OR (MH "Dentition, Secondary")	Expanders - Apply equivalent subjects	Interface - EBSCOhost Research Databases

S9	(MH "Dental Caries")	Expanders - Apply equivalent subjects	Interface - EBSCOhost Research Databases

S8	(MH "Mouth Diseases+") OR (MH "Mouth Neoplasms+")	Expanders - Apply equivalent subjects	Interface - EBSCOhost Research Databases

S7	(MH "Tooth Diseases+") OR (MH "Tooth Injuries+")	Expanders - Apply equivalent subjects	Interface - EBSCOhost Research Databases

S6	(MH "Dental Plaque")	Expanders - Apply equivalent subjects	Interface - EBSCOhost Research Databases

S5	(MH "Periodontal Diseases+") OR (MH "Periodontitis+") OR (MH "Periodontal Cyst") OR (MH "Periodontal Abscess") OR (MH "Chronic Periodontitis")	Expanders - Apply equivalent subjects	Interface - EBSCOhost Research Databases

S4	(MH "Gingivitis+") OR (MH "Gingival Diseases+") OR (MH "Gingival Neoplasms")	Expanders - Apply equivalent subjects	Interface - EBSCOhost Research Databases

S3	(MH "Dental Care+") OR (MH "Dental Care for Disabled") OR (MH "Dental Care for Children") OR (MH "Dental Care for Older Persons") OR (MH "Dental Care for Persons with Chronic Illnesses")	Expanders - Apply equivalent subjects	Interface - EBSCOhost Research Databases

S2	(MH "Oral Hygiene+") OR (MH "Dental Hygiene")	Expanders - Apply equivalent subjects	Interface - EBSCOhost Research Databases	14,439

S1	(MH "Oral Health")	Expanders - Apply equivalent subjects	Interface - EBSCOhost Research Databases

## **Supplementary Table 3: Exclusion reasons**

| **Study** | **Reason for Exclusion** |
| --- | --- |
| Alani et al. (2009) | Wrong population |
| Alfano (2012) | Wrong population |
| Almog et al. (2005) | Wrong population |
| Aquino et al. (2015) | Wrong population |
| Beck et al. (2016) | Wrong population |
| Bernal (2005) | Wrong population |
| Boyleston & Collins (2012) | Wrong population |
| Brennan & Crittenden (2023) | Wrong population |
| Buley (2018) | Wrong population |
| Cermak et al. (2015a) | Wrong population |
| Clough et al. (2016) | Wrong population |
| de Boer et al. (2024) | Wrong population |
| Dickinson (2012) | Wrong population |
| Eachempati et al. (2018) | Wrong population |
| Fenning et al. (2023) | Wrong population |
| Ferguson et al. (2020) | Wrong population |
| Ferreira et al. (2018) | Wrong population |
| Fotedar et al. (2018) | Wrong population |
| Fried (2013) | Wrong population |
| Geggie et al. (1999) | Wrong population |
| Genco & Genco (2014) | Wrong population |
| Gianfrancesco et al. (2016) | Wrong population |
| Goldstein et al. (2008) | Wrong population |
| Haning & Makic (2020) | Wrong population |
| Hansen & Jakobsen (2010) | Wrong population |
| Harmon & Grech (2020) | Wrong population |
| Horne et al. (2015) | Wrong population |
| Israel & Scrivani (2000) | Wrong population |
| Kaintura et al. (2024) | Wrong population |
| Keating et al. (2015) | Wrong population |
| Komulainen et al. (2012) | Wrong population |
| Manuja et al. (2011) | Wrong population |
| McSwiney et al. (2017) | Wrong population |
| Meropol et al. (2014) | Wrong population |
| Moore et al. (2020) | Wrong population |
| Ng et al. (2012) | Wrong population |
| Nishimura et al. (2006) | Wrong population |
| Nowjack-Raymer (1995) | Wrong population |
| O'Gara et al. (2023) | Wrong population |
| Perillo et al. (2018) | Wrong population |
| Planerova et al. (2018) | Wrong population |
| Prado et al. (2022) | Wrong population |
| Ringash et al. (2018) | Wrong population |
| Rogers et al. (2000) | Wrong population |
| Steer (2016) | Wrong population |
| Sullivan & Morgan (2013) | Wrong population |
| Taylor et al. (2014) | Wrong population |
| Tovani-Palone et al. (2024) | Wrong population |
| Tsai et al. (2015) | Wrong population |
| Uchimura et al. (2001) | Wrong population |
| Vérin et al. (2024) | Wrong population |
| Zhou et al. (2019) | Wrong population |
| **Study** | **Reason for Exclusion** |
| Beck et al. (2014) | Wrong outcomes |
| Castaño Novoa et al. (2024) | Wrong outcomes |
| Cermak et al. (2015b) | Wrong outcomes |
| Fox et al. (2021) | Wrong outcomes |
| Greene & Bertagna (2019) | Wrong outcomes |
| Hajdu et al. (2017) | Wrong outcomes |
| Hockstein et al. (2004) | Wrong outcomes |
| Holsonback et al. (2026) | Wrong outcomes |
| Merkel-Walsh & Overland (2024) | Wrong outcomes |
| Muhlböck et al. (2023) | Wrong outcomes |
| Othman et al. (2011) | Wrong outcomes |
| Sethi (2018) | Wrong outcomes |
| Stein et al. (2012) | Wrong outcomes |
| Tan et al. (2024) | Wrong outcomes |
| Yuen (2013) | Wrong outcomes |
| Yuen & Kumar (2015) | Wrong outcomes |
| Chan (2010) | Wrong setting |
| Clayton et al. (2017) | Wrong setting |
| Lloyd-Morris et al. (2020) | Wrong setting |
| Rogers et al. (2011) | Wrong setting |
| Ropke et al. (2021) | Wrong setting |
| Vaughan et al. (2022) | Wrong setting |
| Ajwani et al. (2017) | Limited OT-specific data |
| Bagdesar et al. (2025) | Limited OT-specific data |
| Baxter et al. (2024) | Limited OT-specific data |
| Bonwell et al. (2014) | Limited OT-specific data |
| Jo et al. (2025) | Limited OT-specific data |
| Nusser-Müller-Busch & Lehmann (2020) | Limited OT-specific data |
| O'Malley et al. (2020) | Limited OT-specific data |
| Sanders et al. (2021) | Limited OT-specific data |
| Brighenti et al. (2023) | Wrong population and scoping review |
| Como et al. (2020) | Eligible primary empirical research missing |
| Sachse & Jacob (2024) | Editorial |

## **Supplementary Table 4: JBI Quality Assessment of included studies**

**JBI Critical Appraisal Checklist for Qualitative Research**

| **Studies** | 1. Is there congruity between the stated philosophical perspective and the research methodology? | Is there congruity between the research methodology and the research question or objectives? | Is there congruity between the research methodology and the methods used to collect data? | Is there congruity between the research methodology and the representation and analysis of data? | Is there congruity between the research methodology and the interpretation of results? | Is there a statement locating the researcher culturally or theoretically? | Is the influence of the researcher on the research, and vice- versa, addressed? | Are participants, and their voices, adequately represented? | Is the research ethical according to current criteria or, for recent studies, and is there evidence of ethical approval by an appropriate body? | Do the conclusions drawn in the research report flow from the analysis, or interpretation, of the data? |
| --- | --- | --- | --- | --- | --- | --- | --- | --- | --- | --- |
| Curtin et al., 2023 | Unclear | Yes | Yes | Yes | Yes | Unclear | Yes | Yes | Yes | Yes |
| Junnarkar et al., 2022 | Yes | Yes | Yes | Yes | Yes | Unclear | Unclear | Yes | Yes | Yes |
| Grönbeck Linden et al., 2017 | Unclear | Yes | Yes | Yes | Yes | Unclear | N/A | Yes | Yes | Yes |

## **Supplementary Table 5: GRADE-CERQual Summary of Qualitative Findings**

| **Review finding** | **Studies contributing** | **Methodological limitations** | **Coherence** | **Adequacy of data** | **Relevance** | **CERQual assessment** |
| --- | --- | --- | --- | --- | --- | --- |
| 1. Knowledge of OTs on oral health care — OTs vary in their understanding of oral conditions, sensory-related habits, person-related factors, and the complexity of oral hygiene as a multi-system task | Curtin et al., 2023  Junnarkar et al., 2022  Grönbeck Linden et al., 2017 | No/minor concerns — all 3 studies scored ≥70% on JBI; gaps limited to reflexivity/philosophical positioning | Minor concerns — Curtin reports OTs as having limited knowledge, while Junnarkar reports more developed, specific knowledge (fluoride, sensory triggers); difference plausibly explained by population/context (acute stroke vs. paediatric ASD) rather than true contradiction | Minor concerns — data are rich (direct quotes across all 3 papers) but the finding rests on only 3 primary studies, so the range of settings is narrow | Minor concerns — partial relevance; populations differ substantially (stroke inpatients, community-dwelling elderly, children with ASD), so knowledge content is context-specific even though the pattern (variable OT oral health literacy) is consistent | Moderate confidence |
| 2. Current practices of OTs (assessment, environmental modification, education, sensory-based approaches) | Junnarkar et al., 2022  Grönbeck Linden et al., 2017 | No/minor concerns | No/minor concerns — the two studies describe complementary, non-contradictory practice domains | Moderate concerns — only 2 of 3 studies contribute; Curtin's stroke-unit OTs are not described as having a defined "practice" repertoire, thinning the evidence base | Moderate concerns — findings largely reflect paediatric ASD and elderly community contexts; transferability to acute/inpatient settings (Curtin) is unclear | Moderate confidence |
| 3. Perceived enablers (person-related understanding, knowledge/training, family-centred practice, multidisciplinary support, resources/tools) | Curtin et al., 2023  Junnarkar et al., 2022  Grönbeck Linden et al., 2017 | No/minor concerns | No/minor concerns — enablers reported are consistent in direction across all 3 studies | No/minor concerns — well-supported by rich, multi-source quotations across all subthemes | Minor concerns — "multidisciplinary support" and "family-centred practice" subthemes are strongly grounded in only 1–2 studies each (predominantly Junnarkar), limiting cross-context generalisability | Moderate to high confidence |
| 4. Perceived barriers (oral health literacy, person-related challenges, insufficient disease knowledge, systemic barriers, resource limitations) | Curtin et al., 2023  Junnarkar et al., 2022  Grönbeck Linden et al., 2017 | No/minor concerns | No/minor concerns — barriers converge strongly across all 3 studies (lack of training/confidence, absent guidelines/referral pathways, resource shortages) | No/minor concerns — the most richly and consistently supported theme, with quotations from all three studies across nearly every subtheme | Minor concerns — systemic/resource barriers are setting-specific (hospital ward vs. community/allied health clinic), so the mechanism of the barrier differs even though the category is shared | High confidence |
